# Pride and prejudice – What can we learn from peer review?

**DOI:** 10.1080/0142159X.2020.1774527

**Published:** 2020-07-06

**Authors:** Helen Le Sueur, Arianna Dagliati, Iain Buchan, Anthony D. Whetton, Glen P. Martin, Tim Dornan, Nophar Geifman

**Affiliations:** a Centre for Health Informatics, Faculty of Biology, Medicine and Health, University of Manchester, Manchester, UK;; b The Manchester Molecular Pathology Innovation Centre, University of Manchester, Manchester, UK;; c Department of Public Health and Policy, Faculty of Health and Life Sciences, University of Liverpool, Liverpool, UK;; d Stoller Biomarker Discovery Centre, University of Manchester, Manchester, UK;; e Division of Cancer Sciences, Faculty of Biology, Medicine and Health, University of Manchester, Manchester, UK;; f Centre for Medical Education, Queen’s University Belfast, Belfast, UK

**Keywords:** Peer review, feedback, sentiment, bias, machine learning, subgroup discovery

## Abstract

**Objectives:** Peer review is a powerful tool that steers the education and practice of medical researchers but may allow biased critique by anonymous reviewers. We explored factors unrelated to research quality that may influence peer review reports, and assessed the possibility that sub-types of reviewers exist. Our findings could potentially improve the peer review process.

**Methods:** We evaluated the harshness, constructiveness and positiveness in 596 reviews from journals with open peer review, plus 46 reviews from colleagues’ anonymously reviewed manuscripts. We considered possible influencing factors, such as number of authors and seasonal trends, on the content of the review. Finally, using machine-learning we identified latent types of reviewer with differing characteristics.

**Results:** Reviews provided during a northern-hemisphere winter were significantly harsher, suggesting a seasonal effect on language. Reviews for articles in journals with an open peer review policy were significantly less harsh than those with an anonymous review process. Further, we identified three types of reviewers: nurturing, begrudged, and blasé.

**Conclusion:** Nurturing reviews were in a minority and our findings suggest that more widespread open peer reviewing could improve the educational value of peer review, increase the constructive criticism that encourages researchers, and reduce pride and prejudice in editorial processes.

## Introduction

Peer review is a very influential type of feedback. It educates authors by telling them why their work is not deemed publishable or by recommending improvements, upon which acceptance for publication depends. Peer review can make or break PhDs-by-publication and influence academic career progression. It shapes the field by determining what it is acceptable for authors to say in print and is an arbiter of academic quality. Given its formative place in the education of academically inclined doctors, it is important to evaluate peer review and consider whether there is scope for reshaping this aspect of our field.

Practice points

- Peer review is an influential type of feedback, but not without flaws.
- We identified several factors, unrelated to research quality, that may influence sentiment and language in peer review.
- Using a data-driven approach, we identified three types of reviewers: nurturing reviewers, begrudged, unhelpful reviewers, and blasé, indifferent reviewers.
- More widespread open processes could improve the educational value of peer review.

The reliability of peer review has been questioned (Fiona Godlee 2000). Despite the influence it exerts, a former editor judged it to be so susceptible to flaws that he wrote: ‘If peer review was a drug it would never be allowed onto the market’ (Smith 2010). A Cochrane review published in 2007 found little empirical evidence to show it could assure the quality of biomedical research (Jefferson et al. 2007). Peer review does not prevent inaccurate or specious findings from being published, allowing a significant volume of false, un-reproducible research output to make it to print (Ioannidis 2005). In an experiment conducted by the BMJ, in which eight errors were deliberately inserted into a 600-word paper and then sent to 300 reviewers, the median number of errors spotted was just two (Schroter et al. 2008). Twenty per cent of reviewers spotted none. The development of electronic tools to assess research misconduct and paper retractions (Integrity TCFS 2019) has highlighted that manuscript with significant faults can be passed by peer reviewers and editors. Further, multiple studies have shown that agreement between reviewers on whether a paper should be published is little higher than would be expected by chance (Lock 1985).

Bias is another, significant, problem. For example, an author’s institutional affiliation or prestige may significantly influence review and editors’ final recommendations. One study demonstrated that changing the authors’ names and institutions to less prestigious or well-known ones resulted in 8 out of 12 previously accepted papers being rejected by the journals that had originally published them (Peters and Ceci 1982). The reasons given for rejection were mostly ‘poor quality’. Other author characteristics, such nationality, language, and gender, may also introduce bias from reviewers (Lee et al. 2013). Further, it is likely that peer review suffers from confirmatory bias, where there is a tendency to emphasize and believe experiences that support one’s views and to ignore or discredit novel or radical ideas (Mahoney 1977; Smith 2010). Many organisations ask their staff to undertake unconscious bias training but the same does not apply to reviewers.

Peer review is easily abused; reports of ideas being lifted or competitors’ publication deliberately delayed are common. A form of poor conduct that calls into question the educational value of peer review is the putting down of peers through overly harsh or non-constructive, even contemptuous reviews. This type of ‘meanness’ hurts researchers at all levels of seniority but is particularly troublesome for students or early career researchers, where rejection and discourteous reviews can lead to demotivation and disengagement with research. Receiving an unfair, unkind, or rudely-worded assessment of one’s work is so common that humorous social media accounts, such as ‘Shit My Reviewers Say’ (@YourPaperSucks on Twitter with a following of 48.3 K), collect and disseminate the finest examples. The Facebook group, ‘Reviewer 2 Must Be Stopped!’, shares the poor experiences with the peer review process of nearly 13 K members; and a famous scene from the WWII drama *Der Untergang* (2004), in which Hitler abreacts to his generals telling him Germany is about to be defeated, was spoofed as a rant over one peer reviewer. It would be disturbing if ‘a 19^th^ century gloves off approach’ (Walsh 2005) was still a dominant way of giving feedback to authors over 30 years after Pendleton and colleagues wrote that ‘feedback which only emphasises the learner’s failures or omissions … is all too frequent; the legacy of medical education and of academic life’. Negative feedback, they pointed out, ‘leads to the learner defending what he (sic) did’ rather than learning new ways of working (Pendleton et al. 1984).

The purpose of this study was to help medical researchers and those who educate them, appraise the formative value of peer review. We set out to evaluate the content of peer reviews systematically and objectively, examining factors that influence the language of peer review reports. We took a data-driven approach to identify different types of scientific reviewers in the biomedical domain. 

## Methods

### Data collection – open peer review journals

Peer review reports were downloaded from 10 randomly selected BioMed Central (BMC) journals that operate an open peer review policy. The 10 journals spanned the fields of medical education, medicine, biology, biomedical data mining, public health and infectious disease (see Table 1). Selected journals represented different levels of impact, with impact factors ranging from 1.9 to 7.2.

**Table 1.** Characteristics of the reports evaluated in this study.

Journal	Biodata mining	BMC Cancer	BMC Geriatrics	BMC Medical Education	BMC Medical Ethics	BMC Medical Genomics	BMC Medicine	BMC Public Health	Environmental Health	Infectious diseases	Anonymous peer review
CiteScore 2016	1.53	3.56	2.82	1.71	1.74	2.96	6.81	2.54	3.71	2.9	
Number of review reports	34	64	65	66	63	50	73	61	60	60	46
Number of comments	6 (9.75)	4 (5)	7 (10)	4 (5.75)	6 (7)	7 (5.75)	7 (6)	7 (9)	7 (6.25)	7 (6)	9.0 (5.8)
Number of authors:	4 (2)	8 (4)	6 (3)	5 (2)	4 (2)	7 (3.75)	10 (7)	6 (3)	7 (4)	7 (5)	NA
Days between submission and publication	164.5 (143.75)	251.5 (149.75)	226 (76)	314 (133.5)	203 (141)	194 (159.5)	140 (59)	218 (179)	169 (63)	196 (124.75)	NA
Days between submission and review	46 (82.25)	86 (96)	75 (58)	74 (82.75)	61 (48)	58.5 (80.25)	30 (20)	57 (54)	44.5 (32)	53.5 (63.75)	NA
Request for additional analysis (%)	10 (29.41)	17 (26.56)	28 (43.08)	11 (16.67)	5 (7.94)	4 (8)	12 (16.44)	4 (6.56)	33 (55)	3 (5)	NA
Length of review	2 (2)	4 (1)	4 (1)	3 (1)	5 (1)	2 (2)	2 (2)	3 (1)	5 (1)	2 (2)	2
Level of detail	2 (2)	2 (2)	3 (1)	3 (1)	3 (2.5)	2 (2)	2 (2)	3 (1)	3 (2)	2 (2)	2
Constructiveness	2 (1)	3 (2)	3 (1)	3 (1)	3 (3)	1 (1)	2 (1)	4 (1)	3 (1.25)	2 (1)	1
Positiveness	2 (1)	2 (2)	3 (2)	3 (1)	3 (1)	1 (1.75)	2 (2)	3 (1)	3 (2)	1 (2)	1
Harshness	0 (0.75)	0 (1.25)	0 (0)	0 (1)	0 (1)	1.5 (2)	1 (2)	0 (1)	0 (1)	0 (0)	1
Total number of reviewers	2 (0)	2 (1)	2 (0)	2 (0.75)	2 (0)	2 (1)	3 (1)	2 (0)	2 (0)	2 (0)	3

All the variables are reported as median (IQR), Request for additional analysis is number of positive observations and percentage of the total number of reports.

We randomly selected papers from across the 10 BMC journals (with approximately equal numbers from each), with a given paper included in the analysis if it met the following criteria:

- Published in 2017
- Research article
- Peer review reports were available online

We aimed to collect an even distribution of reviews from papers published in each of the calendar months.

A full description of the data collection process is available in Supplementary File 1.

### Data collection – Anonymous peer reviews

To collect additional review reports from journals without an open peer review policy, we contacted colleagues and collaborators (past and current) to request reviewers’ reports from their own manuscripts. As before, only reports of papers that were eventually published and in the fields of medicine or biology (or related fields) were accepted. We did not restrict the collection to papers published in a specific year, instead we asked for reviews of recently accepted published work. To minimize bias in report selection by our colleagues, the request did not reveal our hypothesis and only gave a general description of the study. A total of 46 reviews from 18 papers were included in this ‘anonymous report’ set; this sample size is based solely on response rates from our colleagues and collaborators.

### Evaluator assessment

We randomly allocated a subset of the 10 BMC journals among ourselves to evaluate. Each evaluator randomly selected a paper from the journal’s website (according to the aforementioned criteria) and extracted the following information from the original (i.e. 1st round) reviewer report for each reviewer individually:

- Paper title, number of authors, original submission date and subsequent publication date
- Date of the 1st review
- Reviewer number
- Level of detail (from 0 to 5)
- Number of comments (from 0 to 5)
- Request for an additional analysis (yes/no)
- Constructiveness (from 0 to 5)
- Positiveness (from 0 to 5)
- Harshness (from 0 to 5)
- Number of rounds of review and which reviewer requested these
- Any quotes that included recurring phrases across peer reviews.

This process was repeated for the anonymous peer reviews.

### Between-evaluator agreement analysis

To assess degree of agreement between evaluators, reviewers’ reports from an additional 10 randomly selected papers (one from each BMC journal) were evaluated and scored by the evaluators. Evaluators’ agreement, for each of the ‘level of detail’, ‘constructiveness’, ‘positiveness’ and ‘harshness’ measures, were assessed by calculating the intra-class correlation coefficient (ICC). ICC estimates and their 95% confidence intervals were calculated based on an absolute-agreement, two-way random-effects model, to indicate the scoring agreement between the four evaluators. The between-evaluator agreement on ‘request additional analysis’ was evaluated with Fleiss’ extended Kappa statistic.

### Regression analysis

To study the risk of getting a harsh review, we performed a regression analysis in which the outcome was defined as the harshness score assigned to each review. Predictors included: the season, the day of the week on which the review was received by the editor, and the number of authors of the manuscript. To control for imbalances of variables, we added evaluator and journal terms in the model.

### Latent class analysis

To identify polytomous classes of reviewers, we applied latent class analysis. This is an unsupervised statistical method for identifying unmeasured class membership among subjects using observed categorical and/or continuous variables (Formann 1992; Linzer and Lewis 2011), used in many studies to stratify disease populations (Keel et al. 2004; Kim et al. 2016; Molgaard Nielsen et al. 2017; Zador et al. 2019). With this analysis, we aimed to identify possible unobserved groups of reviewers based on the following information: level of detail, length of review, number of comments, request for additional analysis, positiveness, harshness, whether the reviewer requested the second round of reviews, and the days between the submission date and the date the review was received by the editor. We excluded highly correlated variables (constructiveness, and if the reviewer requested the third or fourth round of review) and tested the latent class model for 1–10 classes. To identify the optimal number of latent classes we compared the results using the Bayesian Information Criterion (BIC), and selected the model with the lowest BIC. At model convergence, a posterior probability of membership of the latent classes was calculated for each reviewer’s report, which was then assigned exclusively to the class for which the highest posterior probability was obtained. This class-assignment was used for the subsequent characterisation of the classes.

### Statistical analyses

Significant differences between the identified latent classes were tested using Chi-squared tests for categorical or binary variables (length of review, level of detail, constructiveness, positiveness, harshness, and request for additional analysis) and analysis of variance for continuous variables (number of comments and days between submission and review). A chi-squared test was also used to test for significant differences in how reviewers were represented between classes.

## Results

### Evaluations of reviewers’ reports

We evaluated a total of 596 reviewers’ reports from 278 peer-reviewed papers, across 10 BMC journals. Additionally, we included 46 reviews from 18 papers that were published in journals with an anonymous peer review policy. Table 1 presents the characteristics of reviewer reports evaluated in this study. Across the 10 papers reviewed by all evaluators, there was good between-evaluator agreement in the measures extracted from the peer review reports. Agreement was highest for the ‘level of detail’ measure (ICC: 0.585, 95% CI [0.370, 0.766], *p* < 0.001), and lowest for the ‘constructiveness’ measure (ICC: 0.279, 95% CI [0.065, 0.527], *p* = 0.003), with ‘positiveness’ and ‘harshness’ showing fair agreement (ICC: 0.479, 95% CI [0.146, 0.730], *p* < 0.001; and ICC: 0.439, 95% CI [0.240, 0.650], *p* < 0.001, respectively). Similarly, the between-evaluator agreement for the ‘request additional analysis’ indicated fair agreement (Fleiss’s Kappa: 0.336), which was significantly better than no agreement (*p* < 0.001).

### Harshness does not correlate with constructiveness

Examination of correlations between the different factors assessed by our evaluators revealed that, intuitively, harshness negatively correlated with positiveness (*R* = –0.476, *p* < 0.001) and with constructiveness (*R* = –0.16, *p* < 0.001). On the other hand, constructiveness strongly correlated with the level of detail within a given review (*R* = 0.77, *p* < 0.001, Supplementary File 2). Unsurprisingly, the level of detail, number of comments and length of review all positively correlated with each other (*R* > 0.4).

### Seasonal effects on reviewers’ attitude

**Figure 1.**
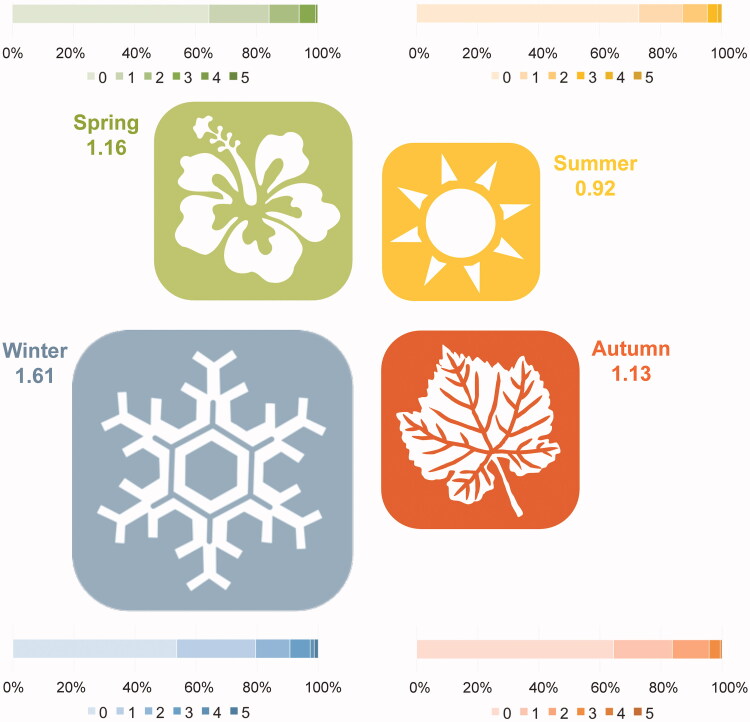
Harshness in reviewers’ reports as a function of the seasons of the year. For each season, the icons size is scaled according to the mean values of harshness in that season (reported alongside it), and a bar plot with the percentage of reviews with each score of harshness (1–5) is provided.

Our regression analysis revealed that seasonality was a predictor of harshness in reports. Reviewer reports submitted during northern hemisphere wintertime were significantly meaner than those submitted any other time of year (*p* = 0.029; Supplementary File 3 and Figure 1).

### Anonymity

To examine the effect that open peer review policy may have on the sentiment of reviewers’ reports, we included an analysis of data collected from anonymous reports. As described above, a total of 46 anonymous reports were obtained from 18 papers. Overall, anonymous reports tended to be harsher, less positive and not as constructive as those published alongside the manuscripts (Supplementary File 4; *p* < 0.001).

### The three types of reviewers

**Figure 2.**
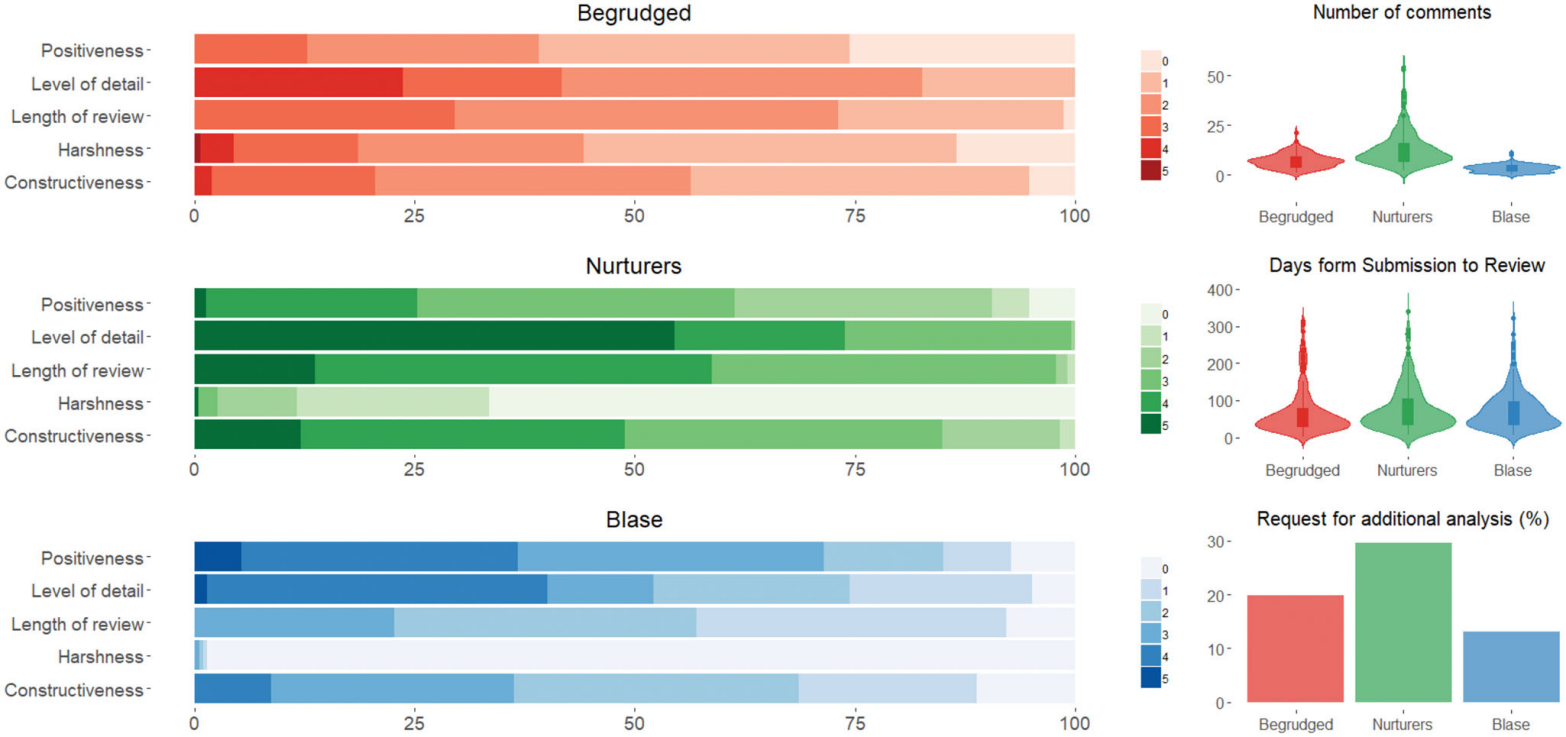
The three subtypes of peer reviewers. These subtypes differed in terms of levels of: positiveness, detail, constructiveness and harshness; and in length of reviews. Further, differences were found in: number of comments, days from submission of the manuscript to review and whether they requested additional analyses to be carried out.

Taking a data-driven approach, and applying machine learning for clustering and stratification, we examined whether distinctive subtypes of reviewers could be identified from reviewers’ reports. We further examined whether these subgroups were associated with distinguishing characteristics. Using latent class analysis, we identified three types of reviewers (Figure 2). The first subtype, ‘the nurturing’, consisted of 22.3% of reviewers, and reflected reviewers who responded quickly and gave longer, more detailed reports which overall were more constructive and positive. The second subtype, ‘the begrudged’, consisted of 33.4% of reviewers, characterised by shorter and less detailed reports that were less constructive and most harsh. Reviewers of this type were less frequent in second rounds of review and less inclined to request additional experiments or analyses. The third subtype, ‘the Blasé’ group, reflected those reviewers who were quickest to submit their reviews, provided shorter less detailed reports and who were generally positive but not very constructive. These made up 44.3% of reviewers.

## Discussion

The principal finding of this research is that positive, constructive feedback, which was not harsh, was the exception rather than the rule. It is worrying that the comparison between open and anonymous peer review reports demonstrated that anonymous reports were generally harsher, less positive and less constructive. The persistence of this ‘19th century gloves off approach’ raises important questions about the educational value of peer review given that the arguments for coupling challenge with support has been laid out increasingly persuasively for half a century. Although Knowles’ ‘adult learning principles’ have largely fallen by the wayside (Norman 1999), his advocacy for socially warm learning environments (Knowles 1975) is now canonical in our discipline. Schmidt’s seminal research into problem-based learning, which began around the same time, showed how congruence between teachers and learners could empower learners to think independently (Schmidt 1993). And yet ‘a 19th century gloves off approach’ (Walsh 2005) remains editors’ and reviewers’ dominant contribution. Is education for scholarship fundamentally different from education for practice and, if so, in what ways? It seems more likely that old traditions die hard and that the ‘scholarship of discovery’ remains ignorant of, or is unwilling to accept, profound changes that have taken place in the ‘scholarship of teaching’ (Beattie 2000). The discipline of medical education, by those arguments, is divided against itself. Medical education would surely benefit from its research tradition taking a leaf out of its pedagogic books.

Our finding of a strong association between seasonality and harshness of review, with a northern hemisphere winter most correlated with negative reviews, may be a manifestation of some form of Seasonal Affective Disorder (Rosenthal et al. 1984). While we were unable to identify the geographical location of reviewers, the majority of research papers originate from northern-hemisphere countries (Scimago Journal & Country Rank 2019; Czerniewicz 2015); this study suggests that reviews in the darker months might be harsher than those in summer months. Further work in this area is required before we could advise editors to make allowances for this.

Most reviewers fell into the ‘Blasé’ group, doing the bare minimum and showing little apparent interest in bettering the manuscript/study. One member of this group, for example, just wrote ‘Accept without revision’, which we think is more likely to show a lack of interest than that the paper was above criticism (given this was from a 1st round review). The next most prevalent category of reviewer was the ‘begrudged’; academics lacking positive regard towards fellow scientists. Whilst their criticisms may have been valid, they would have been far more useful if worded more constructively and less discourteously than: ‘this paper strikes me more as a review than original research - there is no set hypothesis tested or original findings.’; ‘I am concerned that this paper may be more confounding than informative’; ‘The first major issue is that the manuscript was poorly written’; and ‘In the Discussion the authors describe that… What did they expect?’. One reviewer wrote ‘… so broad as to be superficial and meaningless: instead of “narrative”, we could just say “pink cloud of general nice stuff” and be just as meaningful’. Our analysis even found one self-identified begrudged reviewer: ‘The *bad reviewer* in me would think that that might be the reason that … is addressed in the text but not calculated in the tables’. Nurturers, the smallest group, did justice to the peer review process. They differed from other groups in showing they understand their role as improving a piece of work by providing in-depth, constructive criticism, and made the effort to do this.

A novel strength of this work was to apply a data-driven unsupervised learning method to identify and stratify subtypes of peer reviewer with different characteristics similar to reviewer types identified anecdotally and from and qualitative studies (Duncan 2016).

The main limitation stems from the likelihood that open peer review policy influences reviewers to be more constructive and to tone down harsh, cynical or very negative comments. We addressed this potential confounder by including analysis of anonymous review reports of papers published in journals without an open peer review policy, collected from our colleagues. Another limitation is the potential bias introduced by focusing only on BMC journals in our main data collection. To mitigate this, at least in part, we collected reviews from different journals in the BMC family, from different fields of research (within the biomedical domain), and with different levels of Impact Factor. However, a more comprehensive assessment of journals across different fields of research is warranted. Similarly, we were only able to study published articles, meaning comments for rejected manuscripts (which may differ from those of accepted articles) are missed from this study, perhaps leading to an underestimation of harshness in peer review reports. As with many text evaluation studies, there were some differences between our evaluators in the way reviewers’ reports were scored. This was particularly apparent given the subjective nature of the measures by which each evaluator scored the reviews.

Our findings have implications for scholarly practice. We suggest that editors should remind reviewers that authors have invested time and effort in their manuscripts and deserve fair, transparent and constructive criticism. It may also be possible that journal editors themselves fall into the three types identified here, or some other groupings; and while this would require a separate investigation, editors should be mindful of their own attitudes when assessing peer review reports. Open peer review policies could help reduce biases and related problems by removing the blanket of anonymity that allows reviewers to abuse their power. Being more accountable might encourage reviewers to carry out more meticulous evaluations and be more careful in their choice of words. It is plausible that reviewers shift between the types we have described on different occasions. Editorial boards might consider mechanisms to encourage and educate reviewers to adopt the ‘nurturing’ type in preference to the other two.

To conclude, our findings underpin the urban legend of three types of reviewer with empirical evidence – a data-driven classification of Nurturing, Begrudging and Blasé reviewers. We also identified the potential of winter blues to generate harsh reviews, but this requires further investigation by exploring the geographical locations of reviewers in larger datasets. We urge editors to adopt open peer review policies to avoid the cloak of anonymity offered by blinded reviewer comments.

## Supplementary Material

Supplemental Material

## Data Availability

All data generated for this study is available upon request.

## Glossary

**Subgroup discovery:** A data mining technique that identifies associations among different instances or variables with respect to a property of interest. (Sumyea Helal; Subgroup Discovery Algorithms: A Survey and Empirical Evaluation. Journal of Computer Science and Technology, volume 31, pages 561–576 (2016))
