# Supplementary material for: Pride and prejudice – What can we learn from peer review?
Source: Med Teach. 2020 Jul 6;42(9):1012–8. doi: 10.1080/0142159X.2020.1774527 (PMC7497287; doi:10.1080/0142159X.2020.1774527)

Supplementary figure 1: Schematic of the data collection process.

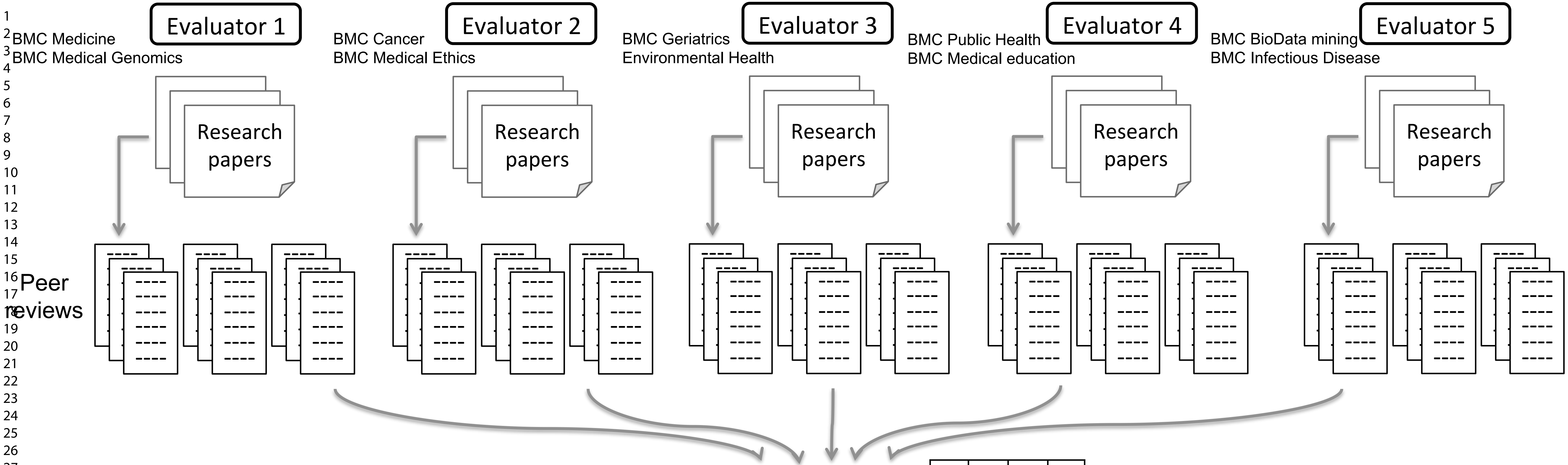

* Score from 1 to 5	1	2	3	...
Level of detail				
Length of review				
No. of comments				
Constructiveness				
Positiveness				
Harshness / meanness				
Request for additional analysis				

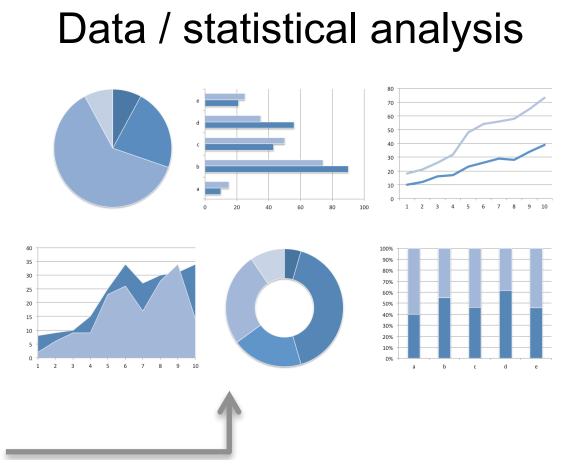

Supplementary figure 2: Correlation plot showing the Person correlation values. Squares with significant p-values (< 0.01) are colored in blue or red accordingly to the correlation value (red if negative, blue if positive), Squares indicating correlations with p-value > 0.01 are white.

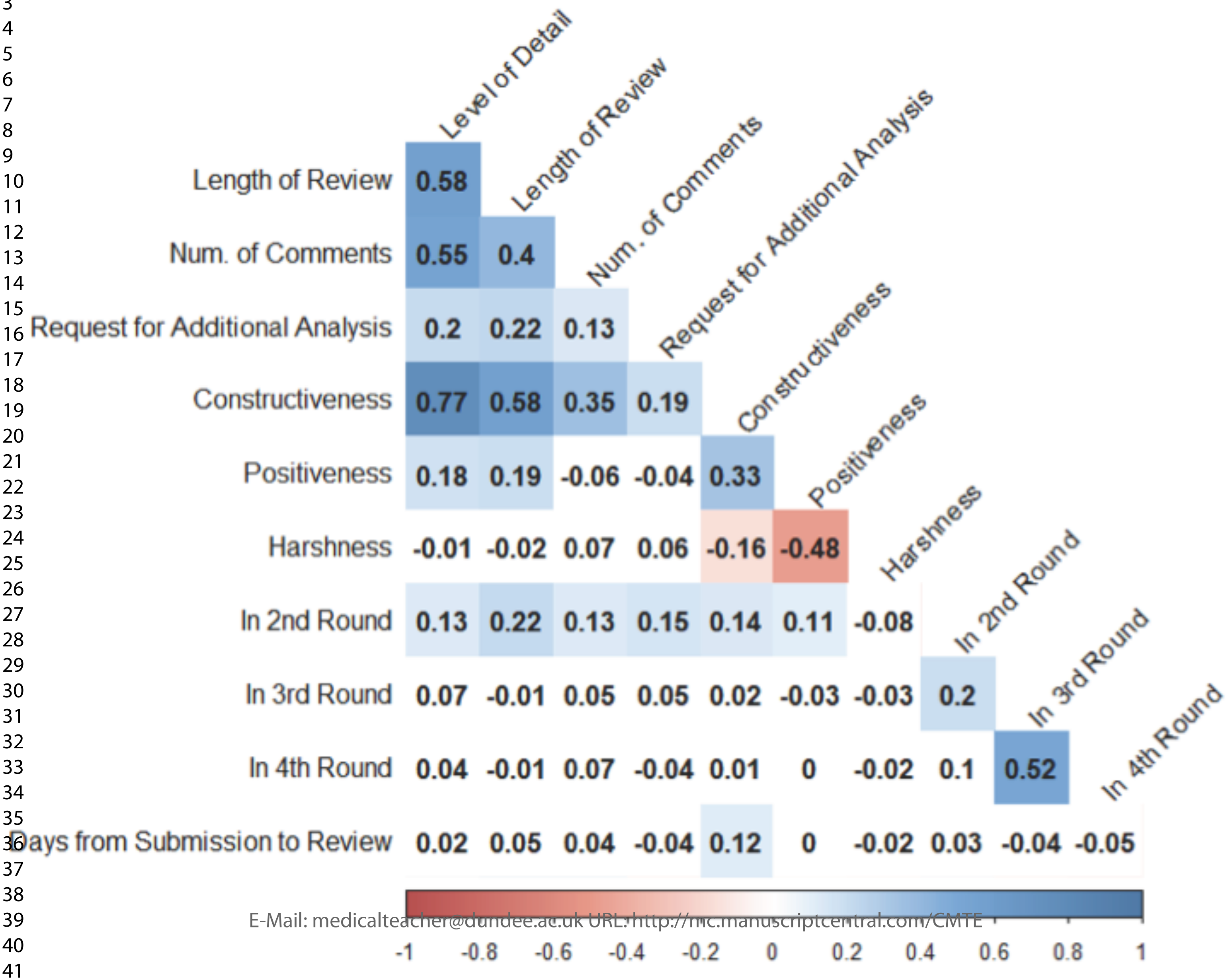

Estimates produced by the regression analysis

	Estimate	*Std.Error*	*t value*	*Pr(>|t|)*
	**Harshness**			
Winter	0.26425	0.08969	2.946	0.00334
Spring	0.11622	0.07355	1.58	0.115
Summer	-0.14819	0.07043	-2.104	0.0358
Fall	-0.10525	0.06786	-1.551	0.121
	**Positiveness**			
Winter	-0.255	0.1166	-2.186	0.0292
Spring	-0.22915	0.12249	-1.871	0.0619
Summer	0.26995	0.1173	2.301	0.0217
Fall	0.18412	0.11309	1.628	0.104

For Peer-Review Only

Supplementary figure 4: A comparison of sentiment in reviewers' reports from open and confidential peer review

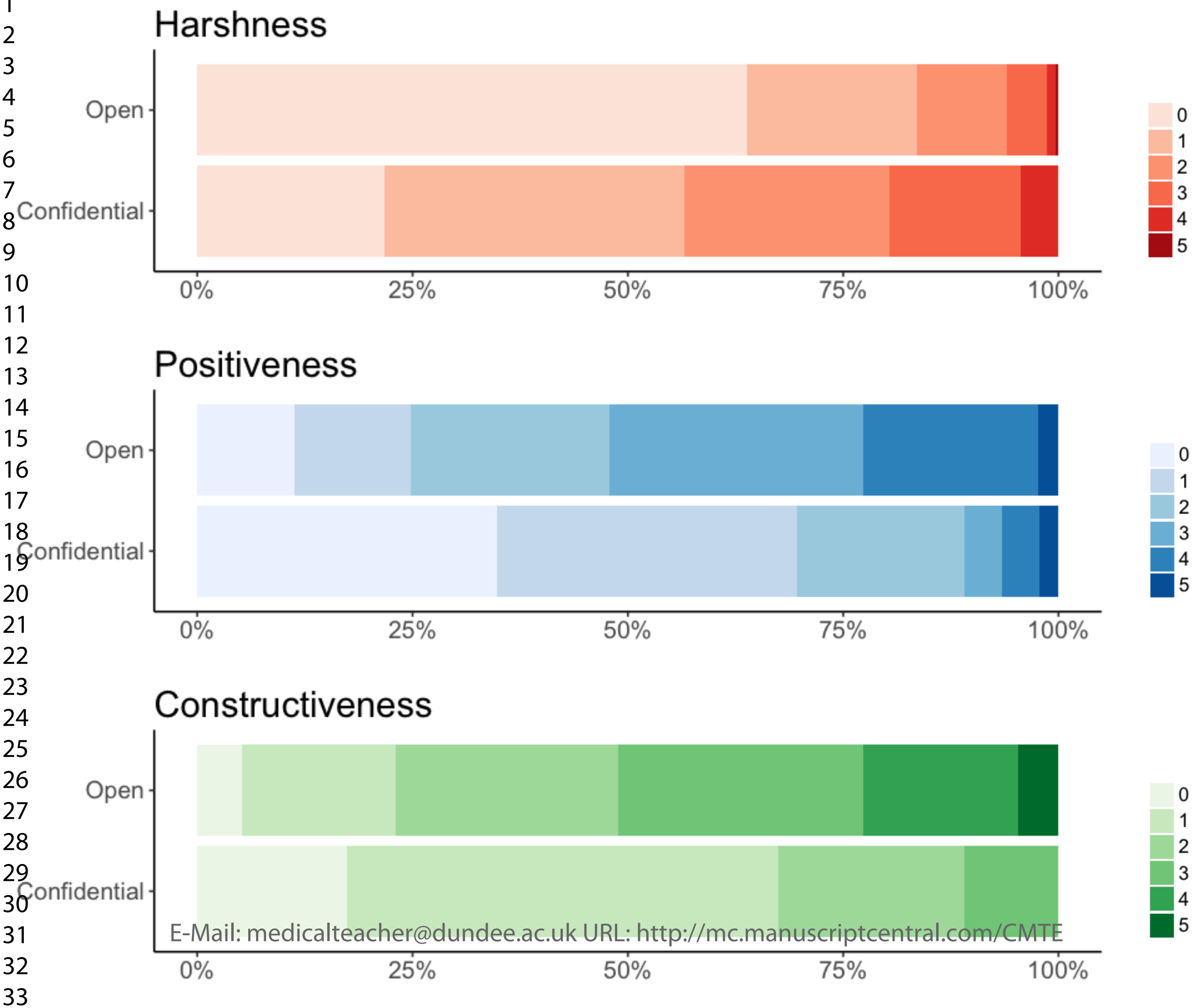

Supplement: Supplemental Material [file IMTE_A_1774527_SM8381.pdf]
